# The Self-Appraisal of Masking Instrument

**DOI:** 10.1186/s42409-022-00032-3

**Published:** 2022-03-21

**Authors:** Ray Block, Eric Plutzer

**Affiliations:** 1 Departments of Political Science and African American Studies, The Pennsylvania State University, State College, USA; 2 Departments of Political Science and Sociology, The Pennsylvania State University, State College, USA

## Abstract

We explore mask-wearing behavior during the coronavirus pandemic using the Self-Appraisal of Masking Instrument (SAMI). We situate this survey-based instrument within a theory in which the decision to mask reflects social identity, an associated identity standard, and appraisals that generate feelings about oneself. Analyses of SAMI’s empirical properties reveal that masking-specific emotional reactions are distinct from emotional reports related to current events and politics (discriminant validity). We also uncover evidence of predictive validity: expressed feelings about masking predict future voting more than 6 months later. We recommend SAMI to researchers interested in studying mask resistance in an increasingly polarized political climate, and the intuition behind SAMI could prove useful in other research contexts in which health decisions reflect a conscious comparison to standards held by those who share an identity or will otherwise pass judgment.

**Supplementary Information:**

The online version contains supplementary material available at 10.1186/s42409-022-00032-3.

## Introduction

The use of simple home-made or surgical masks as a non-pharmacological intervention against the spread of COVID-19 became highly politicized in the USA and continues to be contentious more than a year later. Mask resistance appears unrelated to financial costs or availability. While some citizens report trouble breathing, skin irritation or other discomforts, clinical research shows these to be minor (Rudd & Walsh, 2021) or limited in impact (Bar-On et al., 2021). Rather, masking resistance appears to stem from a complex set of values and attitudes that are shaped by national cultures and within-nation subcultures entwined with group identity and politics.

To better understand mask resistance, we developed two versions of the Self-Appraisal of Masking Instrument, or SAMI. Designed for individuals contemplating masking in the near future, SAMI_Prospective_ asks respondents how they think they will feel if they wear a mask in public. SAMI_Retrospective_ asks individuals who have recently worn a mask in public to report on how they actually felt when donning one. SAMI was designed specifically for use in short polls and surveys administered in English in the USA. The particular emotion words employed in the question stems may require adaptation for use in other cultures and languages. The questions were not intended to be combined into an additive scale. Rather, the questions cover five different self-appraisal considerations, which may be correlated, independent, or complementary.

## Theoretical background

Wearing a mask is more than a private health-related decision. In important ways, the mask becomes a salient component of the self. William James, 1890 (p. 292), argued that the material self was not limited to one’s body, “The old saying that the human person is composed of three parts—soul, body and clothes—is more than a joke.”

Extending that insight, the early sociologist Charles Horton Cooley (1902) coined the term “looking glass self,” meaning that we view ourselves based on how *we believe* others perceive us. Building on work by Burke (1981), Serpe et al. (2020) refined this idea, positing a four-step process of self-appraisal. First, all humans are self-aware of multiple identities—one may be a father, educator, neighbor, Latina, masculine, and so on—and each identity has multiple meanings constituting an *identity standard*.^1^ Second, others also hold ideas about identity standards, creating social expectations. Third, individuals appraise their actions based on how they believe others will judge them—Cooley’s looking-glass-self process. Fourth, if actions are consistent with others’ identity standards, then current behavior is reinforced by positive emotions, such as pride. But if others’ anticipated judgments contradict one’s identity standard, a person will experience negative emotions (such as shame), resulting in pressure to bring self and others’ appraisals back into harmony by changing behavior.

Applied to masks, an individual may initially see no obvious connection between donning a mask and one’s own identity as a Black man, a Republican mother, or any other single or intersectional identity. But if one believes that *others* are making judgments, one will feel pressure to conform with group expectations. Mask wearing may be consistent with an individual’s socially constructed identity standard, inconsistent, or irrelevant. The looking-glass-self model suggests that at least some observed group differences in masking are rooted in this appraisal process. Our goal was to develop questions that would allow respondents to report the kinds of judgments that they believe are being made by others, particularly judgments resulting in pride or embarrassment. The instrument also includes two items related to concerns about personal safety.

## Scale development

### Context: the early months of the COVID-19 pandemic in the USA

We developed the Self-Appraisal of Masking Instruments in the context of confusing and contradictory scientific advice on mask wearing that proliferated in the USA in the first months of 2020 (Chuck, 2020; Eikenberry et al., 2020; Haischer et al., 2020). Experts debated whether SARS-CoV-2 was airborne, whether consumers should eschew surgical masks to ensure adequate supplies for health workers, and whether masks were efficacious for ordinary consumers.

Simultaneously, popular culture in the USA was infused with implicit racial associations of masking with East Asian culture and identity (Ma & Zhan, 2020; Reny & Barreto, 2020; Agius et al., 2020). In addition to incidents reflecting prejudice and suspicion of Americans of East Asian ancestry, other people of color began to report incidents in which they were targeted because of the combination of their race and masking (e.g., Alfonso III, 2020; Baker, 2020; Baptiste, 2020; Block, 2020; Boyd, 2020; Christiani et al., 2021; Vargas & Sanchez, 2020; see also the series of essays in the University of Michigan’s National Center for Institutional Diversity, 2020).

Almost as soon as public health messaging shifted to recommend masking, the willingness to wear them became politicized by then President Donald Trump, who quickly became a lightning rod for an emerging masking debate (Milosh et al., 2020; Sunstein, 2020). Citizens of all political perspectives could not avoid exposure to Trump’s discomfort with and resistance to masking (Utych, 2020; Yamey & Gonsalves, 2020).

There is also a clear gender gap in masking behavior. Women are typically more willing to don them, and for many men the refusal to do so amounts to an act of defiance that can be characterized as a show of performative masculinity (Capraro & Barcelo, 2020; Palmer & Peterson, 2020; Thompson-DeVeaux & Conroy, 2020; Reny, 2020).

It was in this context that we quickly assembled content for a late April poll focusing on pandemic-related topics, guided by presumptions that both identity and personal security shape an individual’s decision to don a mask. Identities influence self-concept by linking actions to feelings such as pride, anxiety, and shame; they move behavior by signaling whether that behavior will enhance how one feels about oneself (Cast, 2003; Carter, 2013; Knowles & Olatunji, 2021). Personal security, in contrast, is rooted in feelings of fear or safety from harm (e.g., Tabernero et al., 2020). Our questions reflecting identity-based concerns were informed by modern versions of Cooley’s notion of the looking glass self.

**Fig. 1:**
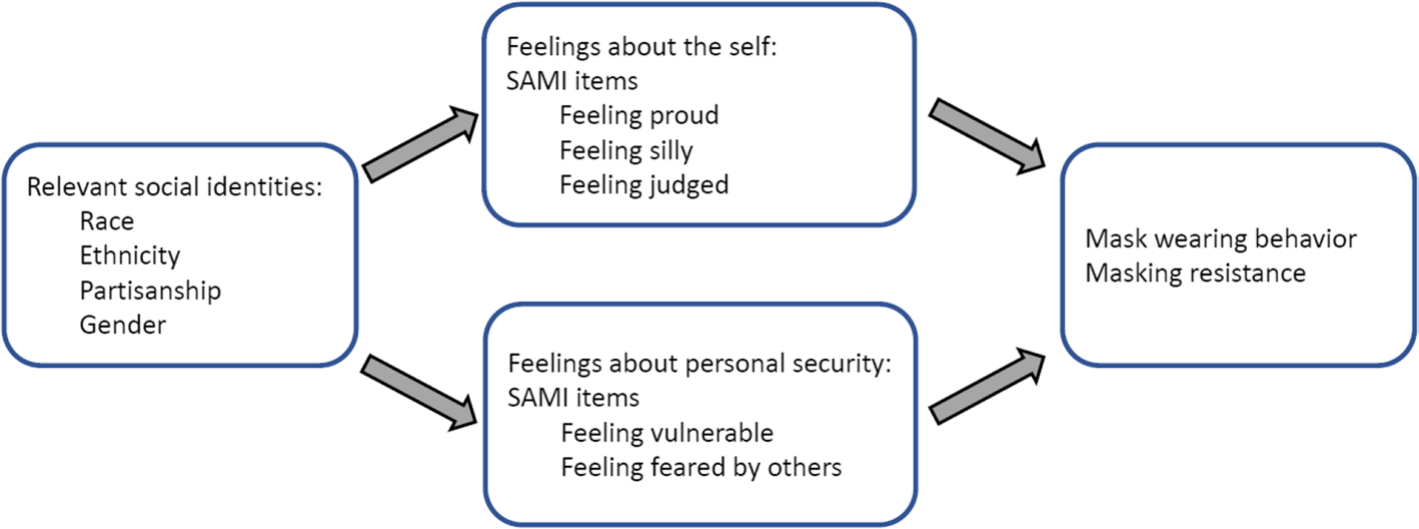
Conceptual framework informing the Self-Appraisal of Masking Scales

Specifically, we were guided by an *identity standard* theory in which the decision to mask up is a signal of identity, an associated identity standard, and appraisals that generate *feelings* about oneself. Figure 1 summarizes the logic underlying our scale development, depicting salient identities in the US context, mediating self-appraisals, and the relevant endpoints.

## Instrument

### SAMI_Prospective_

We designed two SAMI sequences, with assignment determined by a prior screening question on frequency of mask-wearing in the previous week (see Supplementary Materials A2). The prospective version of SAMI was designed for those who did not mask up and who could not provide retrospective reports. Non-wearers are asked “How do you think you *would feel* if you did wear a mask when shopping at a supermarket or commuting to work?” This was followed by five statements that include three identity-relevant appraisals and two related to self-protection and safety:

- I would feel proud I am contributing to stopping the spread of COVID-19.
- I would feel self-conscious that others were judging me.
- I would feel silly.
- I would be fearful that someone might think I was dangerous.
- I would be worried that a mask would subject me to racist aggression.

For each, respondents could answer “Definitely would feel this way,” “Possibly would feel this way,” or “Definitely would not feel this way.” Respondents could skip a question, but a “Do not know” option was not provided.^2^

### SAMI_Retrospective_

Those who wore a mask are asked, “*How did you feel when you did wear a mask?”* followed by a series of statements which were rated on a scale of “Very much,” “Somewhat,” and “Not at all.”

- I felt proud I am contributing to stopping the spread of COVID-19.
- I felt self-conscious that others were judging me.
- I felt silly.
- I was fearful that someone might think I was dangerous.
- I worried that a mask would subject me to racist aggression.

The choice of these items reflected resource constraints (a single screen with a grid of 4–6 questions) and our theoretical interest in both identity-based self-appraisals (proud, judged and silly) and safety-related perceptions (feared, fearful).

## Fieldwork and descriptive statistics

We fielded the SAMI in 27–30 April 2020 as a module in a nationally representative (*N* = 1000) online public affairs poll of US adults conducted periodically by YouGov for Penn State's McCourtney Institute for Democracy. To place our work in context, the United States recorded its one millionth confirmed case, and the 7-day average death rate hit records every day the April poll was in the field. However, much of the nation had been spared. Indeed, 12% of our April wave respondents lived in counties without a single coronavirus-related death; half lived in counties with fewer than five cumulative deaths per 100,000 residents.

We repeated the module, with slight changes in question wording noted below, in 14–18 September 2020 with a fresh sample of 1000 respondents,^3^ by which the USA had recorded over 6½ million cases and 95% of respondents lived in counties with more than five COVID-19 fatalities per 100,000 people. The September version of the retrospective instrument, answered by 94% of respondents, differed slightly from that used in April. The lead-in question was *“How did you feel while wearing a mask?* (“while wearing” rather than “when you did wear”) and the first response was “Strongly” (rather than “Very much”; these changes were intended to make the language clearer and briefer).

We first report on the answers to all five questions, by wave and by version (as a shorthand, all tables and figures use the mnemonics *proud, judged, silly, feared*, and *fearful*, respectively). Table 1 shows that all five items display substantial variation overall, but with important differences both across items and phases.^4^ The only positive self-appraisal, pride, is experienced more frequently than any other among mask wearers. Those who had not worn a mask when interviewed in April show much lower levels of *anticipated* pride and one in five expected to feel very *silly* (in contrast to only ten percent of actual mask wearers); safety-related concerns decrease slightly between April and September.^5^

**Table 1:** Distributions of SAMI items, by phase (column percentages)

	April, never masked (*N* = 240)		April, wore mask (*N* = 760)	September, wore mask (*N* = 963)
***Proud***				
Definitely would not	40.8	Not at all	20.8	24.7
Possibly would	38.8	Somewhat	35.9	25.8
Definitely would	20.4	Very much (strongly)^a^	43.3	49.5
Total	100.0	Total	100.0	100.0
***Silly***				
Definitely would not	51.7	Not at all	68.6	77.4
Possibly would	25.4	Somewhat	22.1	12.5
Definitely would	22.9	Very much (strongly)^a^	9.3	10.2
Total	100.0	Total	100.0	100.0
***Judged***				
Definitely would not	67.9	Not at all	71.7	79.0
Possibly would	22.9	Somewhat	18.8	13.6
Definitely would	9.2	Very much (strongly)^a^	9.5	7.4
Total	100.0	Total	100.0	100.0
***Feared***				
Definitely would not	70.0	Not at all	75.4	84.9
Possibly would	21.7	Somewhat	17.1	9.6
Definitely would	8.3	Very much (strongly)^a^	7.5	5.5
Total	100.0	Total	100.0	100.0
***Fearful***				
Definitely would not	90.0	Not at all	85.5	84.8
Possibly would	5.8	Somewhat	7.5	10.6
Definitely would	4.2	Very much (strongly)^a^	7.0	4.6
Total	100.0	Total	100.0	100.0

^a^In the April wave, mask wearers were offered response options of “Not at all,” “Somewhat,” and “Very much. The last option was “Strongly” in the September wave

## Quality criteria

### Reliability and internal consistency

We created the five items without clear expectations of whether they could be usefully combined into an additive scale. We expected that the positive self-appraisal (pride) would be inversely correlated with negative identity-based appraisals (silly and judged), but we were open to the possibility of weak or complementary associations generally. To explore this, Table 2 reports unweighted pairwise Spearman rank-order correlations among the five items.^6^ The right-hand column reports the “item-to-rest” correlation (the Pearson correlation of each item with a scale created by adding together the standardized scores of the other four items). The results show that the pride item consistently has the lowest correlation with the others: its item-to-rest correlation never exceeds 0.15.

**Table 2:** Pairwise inter-item Spearman correlations (unweighted), by phase

	Proud	Silly	Judged	Feared	Fearful	Item-rest correlation^(a)^
**April, those who never masked (** ***N*** **= 240)**						
I felt proud	1.00					- .14
I felt silly	- .44	1.00				.56
I felt judged	- .04	.48	1.00			.51
Others would fear me	.01	.31	.47	1.00		.46
I worried about aggression towards me	.07	.20	.36	.41	1.00	.33
**April, those who wore a mask (** ***N*** **= 760)**						
I felt proud	1.00					.07
I felt silly	- .12	1.00				.40
I felt judged	.08	.54	1.00			.64
Others would fear me	.08	.39	.53	1.00		.65
Fearful of aggression towards me	.13	.33	.47	.60	1.00	.60
**September, those who wore a mask (** ***N*** **= 964)**						
I felt proud	1.00					- .06
I felt silly	- .32	1.00				.56
I felt judged	- .03	.45	1.00			.59
Others would fear me	.05	.40	.61	1.00		.58
Fearful of aggression towards me	.06	.33	.50	.59	1.00	.52

^a^The item-rest correlation is the Pearson correlation of each item with a scale created by adding together the standardized scores of the other four items

As expected, feelings of pride are inversely related to feeling silly. But interestingly, this negative correlation is highest among non-wearers in April (*ρ* = − 0.44) and in September (*ρ* = − 0.32), but close to 0 among mask wearers in the early phase of the pandemic. This turns out to reflect the increasing degree of partisan polarization of mask wearing—a topic we examine in our ongoing research.

### Discriminant validity

One concern about any novel questionnaire item is that respondents may attend to one or more key words without fully comprehending the intended meaning. Here, salient keywords like “proud” and “fearful” might lead respondents to give answers reflective of their personalities (trait emotion) or current mood (state emotion). Fortunately, the survey included a short emotions battery in which respondents were asked to answer open-ended questions about events in the news or politics that made them feel proud, angry, hopeful, and worried. Each was then followed by a scaler rating in which the respondent told us, for example, how proud the named event made them (full wording in SM A3). If SAMI measures reflect trait or state emotions, then those reporting that masking made them feel proud would also express high levels of pride in recent public events. We thus examined the correlation of the *civic* “how proud” measure with the SAMI pride question, and the *civic* “how worried” measure with the SAMI fearful question. The Spearman correlation of the two pride measures was negligible (*ρ*_*Spearman*_ = 0.05; *p* = 0.045), while the fearful and worried measures were *negatively* correlated, but shy of statistical significance (*ρ*_*Spearman*_ =  − 0.03; *p* = 0.159). These tiny correlations constitute evidence of discriminant validity.

### Predictive validity: future voting

Although data were collected in two independent cross-sections, YouGov panelists are routinely asked how they voted in each recent election. Of the 1000 April respondents, 818 were re-interviewed by YouGov within weeks of the November 2020 election and asked if they voted, and—if so—for whom they cast a presidential ballot. We acquired these reports from YouGov and merged them back to our survey, allowing us to create an April–November panel data set, albeit one with only a single time-2 measure: presidential vote.

**Fig. 2:**
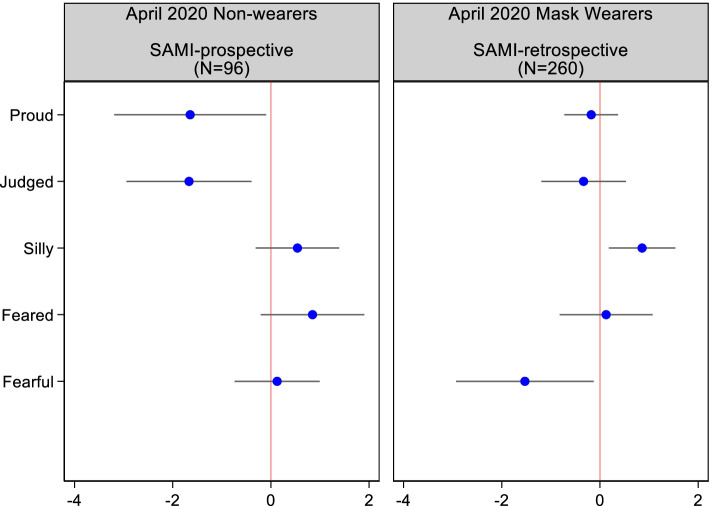
Effects of SAMI appraisals on casting a vote for Donald Trump in the November 2020 election (logistic regression coefficients; restricted to respondents who did not vote for a major party candidate in 2016)

Because Donald Trump politicized the use of surgical masks, we believe a good test of predictive validity would entail seeing whether self-appraisals reported to us in April predict voting for Donald Trump 27 weeks later. Because prior Trump support surely predicts mask appraisals, we restricted our analyses to only voters who did not vote for a major party candidate in 2016: non-voters and minor party supporters. This left us with a sample of 96 April-wave respondents who were not wearing masks and answered the prospective version of SAMI and 260 respondents who completed SAMI_retrospective_. Our dependent variable is coded as 1 if the respondent voted for Trump half a year later and 0 otherwise; each SAMI measure is modeled as a numeric variable.^7^ The binary logistic regression results, summarized in Fig. 2, show some predictive power.

Among those not masking at the time of the April interview, those anticipating that they would feel proud and those who felt they would be judged were far less likely than others to vote for Trump 6 months later. The pattern is quite different among mask wearers. Those who reported having felt silly were much more likely to have been later mobilized to vote for Trump while those who felt fearful about being the target of racist aggression were significantly less likely to do so. While these data are noisy, by excluding previous major party voters, we have narrowed down to those at risk of having their politics and vote impacted by mask mandates and how they felt about themselves in that context. However, the results are quite similar if we include major party voters, with one exception (as shown in Section D of the Supplementary Material, the effect of feeling fearful of others is indistinguishable from zero).

## Conclusion

In this paper, we evaluate the empirical properties of the Self-Appraisal of Masking Instrument. The inspiration for SAMI stems from the contemporary controversy over mask wearing in the era of COVID-19, and we situate SAMI within a theory of social identity standards and masking behavior. We explore the response distributions of the items comprising “prospective” and “retrospective” versions. By analyzing variables presumed by the masking literature to correlate with it, we demonstrate that SAMI has appropriate construct validity. We believe the items comprising SAMI will be useful to survey researchers who explore the link between social identity, self-appraisal, and disease mitigating behavior in the context of the COVID-19 pandemic. With some altering, the prospective and retrospective SAMI can be adapted for non-English languages and cultures. Moreover, researchers might find the ideas gauged by this instrument to be applicable to other contexts: for example, the study of group difference in the willingness of people to receive the COVID-19 vaccine. More generally, SAMI could be adapted for other health behaviors characterized by identity-related resistance, including adolescent resistance to orthodontics (Lewit & Virolainen, 1968; Hamdan, 2004) or the unwillingness of elderly to use assistive devices (walkers) because of their implications for identity (Astell et al., 2020).

The rapid response of this poll to the emerging controversy over masking and the limited space on the poll limited the length of the instrument and the opportunities for pre-testing. Those limitations acknowledged, the instrument shows evidence of masking-specific emotional reactions that are quite distinct from emotional reports related to current events and politics (discriminant validity) and shows evidence of predictive validity: expressed feelings about masking predict future voting more than 6 months later. In that light we believe the SAMI instruments show considerable promise, even as we recognize that the instruments merit further investigation and refinement.

## Supplementary Information

**Additional file 1.**

## Data Availability

Replication data set, codebook, and code to replicate all tables and figures will be made available at https://dataverse.harvard.edu/dataverse/MOTN/ no later than 12 months after publication. Investigators may request access to the data sooner.

## Footnotes

1 The idea of an identity standard is closely related to, but distinct from, the sociological notion of a *role*. The latter is typically understood to reflect social consensus on expected behaviors and responsibilities, but these need not be fused with identity. Someone can enact the socially proscribed roles of parent or student without internalizing those roles as important aspects of the self. In contrast, an identity standard presumes strong identification, internalization, and motivation to display behaviors consistent with expectations — even when the display is only to oneself.

2 In spite of a clear statement on the consent screen that respondents are free to skip any and all questions, we report no missing data. This is typical of online panelists who worry about being denied future participation opportunities for not answering (e.g., Bjornsdottir et al., 2014; Shin et al., 2012).

3 Each of the two independent samples were drawn from YouGov’s nonprobability panel, matched to fit American Community Survey benchmarks on gender, age, race, ethnicity, education and region (see Ansolabehere & Rivers, 2013; Rivers & Bailey, 2009). The target population consists of all permanent resident US adults who can complete a survey in English. YouGov-provided post-stratification weights are employed in all analyses reported in this paper.

4 We also assessed data quality by examining non-differentiation. As detailed in SM section B, we found no evidence of invalid straightlining (Reuning & Plutzer, 2020).

5 Recognizing the difference between *actual* versus *anticipated/hypothetical* mask wearing, we keep these versions of the survey questions separate in our analyses.

6 Table C1 reports the corresponding table with Pearson correlations, Cronbach’s alpha (the lower bound scale reliability), along with the calculated value of alpha for a scale composed of the four negative items (all but pride). Table C2 reports the corresponding polychoric correlations. These show that the Pearson correlations are nearly identical, while polychoric correlations are on average about 50% higher overall but reflect the same patterns.

7 The alternative is to represent each three-category variable by two indicator variables. This will, by necessity, improve the fit of the model but also consume an additional five degrees a freedom. But more fundamentally, treating the SAMI measures as categorical requires fitting data to very small cells. As a general rule, asymptotic efficiency erodes with N’s under 30 and one third of the cells corresponding to dummy indicators have no more than 10 cases. In short, the data are simply too sparse to estimate the corresponding models. Treating these as metric allows the model to draw strength from the full distribution of each, making this the best solution among several less-than-optimal approaches.
