# Supplementary material for: The Self-Appraisal of Masking Instrument
Source: Meas Instrum Soc Sci. 2022 Mar 21;4(1):4. doi: 10.1186/s42409-022-00032-3 (PMC8935613; doi:10.1186/s42409-022-00032-3)

SUPPLEMENTARY MATERIAL FOR

The Self Appraisal of Masking Instrument

Measurement Instruments in the Social Sciences
https://doi.org/10.1186/s42409-022-00032-3

Ray Block Jr
Penn State University
ray.block@psu.edu

Eric Plutzer
Penn State University
plutzer@psu.edu

A. Questionnaire

B. Data quality check: Straightlining

C. Pairwise Inter-item Pearson and Polychoric Correlations

D. Predictive validity: Voting behavior in the 2020 Presidential election predicted by April, 2020, SAMI appraisals (all voters).

A. Questionnaire

A1. SAMI Instruments from April 2020 poll (items used in this paper are highlighted by bold font).

activities_lastweek *Still thinking about the **last week**. Please tell us how often you engaged in the following.*

	Every day	Most days	Once or two days	Never
a. I spent quality time with my family or friends				
b. I watched videos, TV or movies				
c. I listened to music				
d. I played video games				
e. I followed news about the coronavirus pandemic				
f. I participated in a video chat with friends or family				
g. I prayed for people who are sick from the coronavirus				
**h. I wore a mask to protect myself or others**				

If "activities_lastweek_h" (wore a mask) = Never:

mask_would **How do you think you *would feel* if you did wear a mask when shopping at a supermarket or commuting to work?**

	**Definitely would feel this way**	**Possibly would feel this way**	**Definitely would not feel this way**
**a. I would feel proud I am contributing to stopping the spread of Covid-19.**			
**b. I would feel self-conscious that others were judging me.**			
**c. I would feel silly.**			
**d. I would be fearful that someone might think I was dangerous.**			
**e. I would be worried that a mask would subject me to racist aggression.**			

If "activities_lastweek_h" once or more:

mask_did **How did you feel when you did wear a mask?**

	Very much	Somewhat	Not at all
a. I felt proud I am contributing to stopping the spread of Covid-19.			
b. I felt self-conscious that others were judging me.			
c. I felt silly.			
d. I was fearful that someone might think I was dangerous.			
e. I worried that a mask would subject me to racist aggression.			

A2. SAMI Instrument from September 2020 poll.

wore_mask . **In the last week, how many days did you wear a mask to protect yourself or others?**

1. Every day last week.
2. Most days last week.
3. One or two days last week.
4. Not last week, but at least once in the last two months.
5. I have never worn a mask.

[Skip if wore_mask = 5] mask_did   How did you feel while wearing a mask?

	Strongly	Somewhat	Not at all
I felt proud I am contributing to stopping the spread of Covid-19.			
I felt self-conscious that others were judging me.			
I felt silly.			
I was fearful that someone might think I was dangerous.			
I worried that a mask would subject me to racist aggression.			

A3. Items used to assess discriminant validity (Questions Q2 and Q8 from the sequence described below).

whatproud Q1. What **is there [about American politics today/in the news]**[1] that makes you feel proud?

howprowd Q2. You said <<short phrase>> makes you proud. How proud does that make you feel?

4. Extremely proud.
3. Very proud.
2. Somewhat proud.
1. Just a little proud.
0. Nothing made me proud.

whatangry Q3. What **is there [about American politics today/in the news]** that makes you feel angry?

howangry Q4. [Unchanged] You said <<short phrase>> makes you angry. How proud does that make you feel?

4. Extremely angry.
3. Very angry.
2. Somewhat angry.
1. Just a little angry.
0. Nothing made me angry.

[1] Questions Q1 and Q3 have randomized prompts. Fifty percent of respondents are randomly assigned to the "in the news" condition to both Q1 and Q3, while 50% are assigned to the "politics" prompt for both questions.

hopewhat Q5. [Unchanged] Looking ahead, **what makes you most hopeful** about where America is headed **in the next 12 months**?

howhopeful Q6. [Unchanged] You said <<short phrase>> **makes you most hopeful**. How hopeful does that make you feel?

4. Extremely hopeful.
3. Very hopeful.
2. Somewhat hopeful.
1. Just a little hopeful.
0. Nothing made me hopeful.

worrywhat Q7. [Unchanged] Looking ahead, **what worries you most** about where America is headed **in the next 12 months**?

howworried Q8. [Unchanged] You said <<short phrase>> **worries you the most**. How worried does that make you feel?

4. Extremely worried.
3. Very worried.
2. Somewhat worried.
1. Just a little angry.
0. Nothing made me worried.

B. Data quality check: Straightlining

B1. Supplementary text

Straightlining

Valid straightlining can occur whenever the data generating mechanism – including the inherent consistency of the latent attitudes, the amount of random measurement error, and the width of the answer bins – leads effort-expending respondents to give the same sincere answer to all questions in the sequence (Reuning and Plutzer 2020). Under many common conditions, high reliability can even elevate levels of valid straightlining. On the other hand, excessive straightlining can signal high levels of satisficing and low data quality.

We find about 14% of all respondents provided the same answer to all five questions. This is not an unusually high number as four of the items are highly intercorrelated and effortful respondents therefore should provide the similar answer to all four of these. The fifth item, feeling proud, is not genuinely reverse coded (having a strong negative association with only one of the other items). To benchmark straightlining, we also calculated the degree of straightlining for a battery of questions asking about contemporary social, economic and political problems in the same survey. More respondents give the same answer to all of these (18%) and, the likelihood of respondents straightlining the SAMI scale was completely independent of their likelihood of straightlining the problems scale (design-based test of independence: $F(1, 1999) = 2.275$, $p = 0.13$). As a result, straightlining does not seem to be a problem in our data and cannot account for the strong inter-correlations among four of the items.

B2. Items used in straightlining assessment.

For each of the following, please tell us whether you feel this is a serious problem facing the nation today. [Response options were: Very serious problem, somewhat serious, not a problem at all]

- The barriers to voting in person on election day.
- The percentage of the public who lack health insurance.
- The federal minimum wage ($7.25 per hour) being too low.
- The attention that big corporations get from members of Congress and the President.
- The government's capacity to respond to major emergencies.
- American citizens' inability to work together to solve major problems.
- The number of Americans who have always been one paycheck away from a financial crisis.

Tabulation of straightlining on two survey grids (row percentages)

	Gave same answer to all SAMI questions		
Gave same answer to all "problems" questions	No	Yes	Total
No	87.4	12.6	100.0
Yes	83.2	16.8	100.0
Total	86.6	13.4	100.0

Design-based $F(1, 1999) = 2.2746$ $p = 0.1317$

C. Pairwise Inter-item Pearson and Polychoric Correlations .

Table C1. Pairwise inter-item Pearson correlations (unweighted), by phase

April, Those Who Never Masked (N = 240)

	Proud	Silly	Judged	Feared	Fearful	Item-rest correlation [a]
I would feel proud	1.00					-.14
I would feel silly	-.44	1.00				.56
I would feel judged	-.02	.47	1.00			.51
Others would fear me	.00	.31	.49	1.00		.46
I would be fearful of aggression	.05	.20	.34	.41	1.00	.33

April, Those Who Wore a Mask (N = 760)

	Proud	Silly	Judged	Feared	Fearful	Item-rest correlation [a]
I felt proud	1.00					.07
I felt silly	-.11	1.00				.40
I felt judged	.10	.52	1.00			.64
Others would fear me	.10	.41	.58	1.00		.65
Fearful of aggression towards me	.14	.33	.50	.63	1.00	.60

September, all mask wearers (N = 964)

	Proud	Silly	Judged	Feared	Fearful	Item-rest correlation [a]
I felt proud	1.00					-.06
I felt silly	-.34	1.00				.56
I felt judged	-.01	.43	1.00			.59
Others would fear me	.07	.38	.61	1.00		.58
Fearful of aggression towards me	.09	.34	.53	.63	1.00	.52

[a] The item-rest correlation is the Pearson correlation of each item with a scale created by adding together the standardized scores of the other four items.

Table C2. Polychoric correlations (unweighted), by phase

April, Those Who Never Masked (N = 240)

	Proud	Silly	Judged	Feared	Fearful
I would feel proud	1.00				
I would feel silly	-.59	1.00			
I would feel judged	-.02	.62	1.00		
Others would fear me	.02	.44	.73	1.00	
I would be fearful of aggression	.04	.43	.59	.73	1.00

April, Those Who Wore a Mask (N = 760)

	Proud	Silly	Judged	Feared	Fearful
I felt proud	1.00				
I felt silly	-.35	1.00			
I felt judged	.05	.68	1.00		
Others would fear me	.15	.50	.72	1.00	
I worried about aggression towards me	.30	.53	.78	.86	1.00

September, all mask wearers (N = 964)

	Proud	Silly	Judged	Feared	Fearful
I felt proud	1.00				
I felt silly	-.46	1.00			
I felt judged	-.04	.65	1.00		
Others would fear me	.12	.63	.81	1.00	
I worried about aggression towards me	.15	.56	.75	.83	1.00

D. Predictive validity: Voting behavior in the 2020 Presidential election predicted by April, 2020, SAMI appraisals (all voters).

Figure A1. Effects of SAMI appraisals on casting a vote for Donald Trump in the November 2020 election (logistic regression coefficients). All respondents with valid vote report for 2020 election.

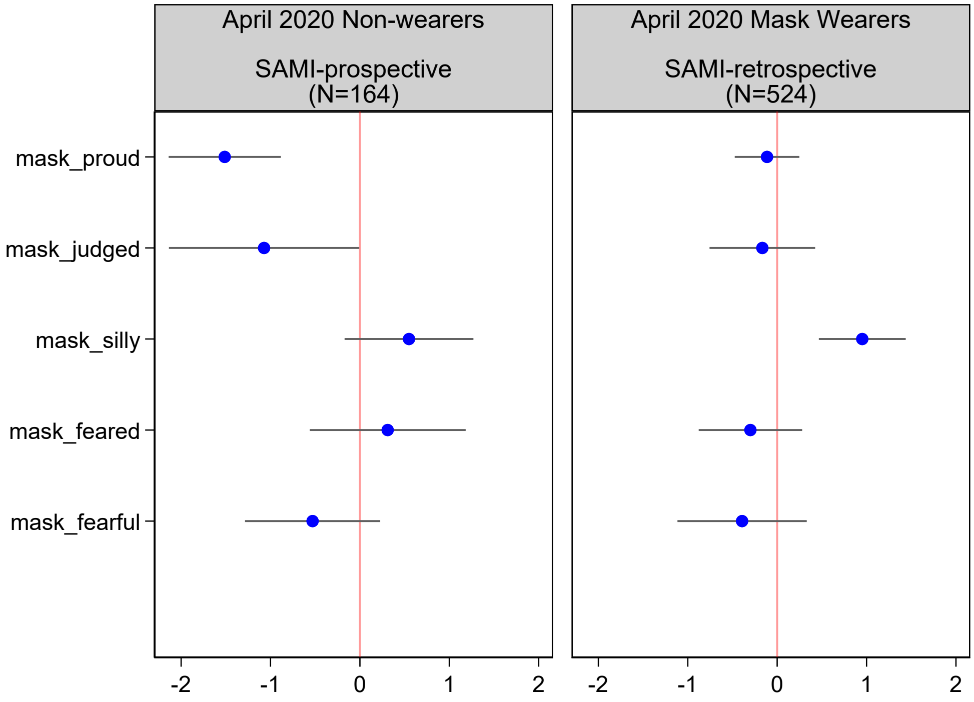

Supplement: Supplementary file 1 — Additional file 1. [file 42409_2022_32_MOESM1_ESM.pdf]
